# Anxiety and depression in women with urinary incontinence using E-health

**DOI:** 10.1007/s00192-020-04227-2

**Published:** 2020-02-24

**Authors:** Nils Hansson Vikström, Elisabet Wasteson, Anna Lindam, Eva Samuelsson

**Affiliations:** 1 Department of Public Health and Clinical Medicine, Umeå University, Umeå, Sweden; 2 Department of Psychology and Social work, Mid Sweden University, Östersund, Sweden

**Keywords:** Urinary incontinence, Depression, Anxiety, eHealth, Epidemiology

## Abstract

**Introduction and hypothesis:**

Previous studies have found high prevalence rates of anxiety and depression in women with urinary incontinence (UI). This study investigates the prevalence in women who had turned to eHealth for treatment of UI and identifies possible factors associated with depression.

**Methods:**

We analyzed data from two randomized controlled trials evaluating eHealth treatment for UI, including 373 women with stress UI (SUI), urgency UI (UUI), or mixed UI (MUI). We used the Hospital Anxiety and Depression Scale (HADS) and defined a score of ≥8 as depression or anxiety. The ICIQ-UI-SF questionnaire was used to score incontinence severity. Logistic regression was used to determine factors associated with depression and anxiety.

**Results:**

Women with UUI or MUI were older than women with SUI, mean age 58.3 vs 48.6 years (*p* = <0.001). Four out of five participating women had a university education. The prevalence of anxiety and depression in women with SUI was 12.4% and 3.2% respectively. In women with MUI/UUI, 13.8% had anxiety and 10.6% had depression. In multivariate analyses, the odds ratio of having depression was 4.2 (95% CI = 1.4–12.3) for women with MUI/UUI compared with SUI when controlling for other risk factors.

**Conclusion:**

The odds of depression in women with MUI/UUI were increased compared with SUI. The prevalence of anxiety and depression was considerably lower than reported in large cross-sectional surveys. Socioeconomic differences may partly explain this finding, as the use of eHealth still is more common among highly educated women.

## Introduction

Urinary incontinence (UI) is defined as any involuntary leakage of urine among adults and about 1 in 4 women are affected [1, 2]. The most common type is stress urinary incontinence (SUI), but urgency incontinence (UUI) and mixed type incontinence (MUI) are also widespread. The prevalence of UI varies from study to study, but in general, UI increases with age from about 20–30% in young adult life to about 30–50% in the elderly population [3]. Questionnaires about history, bladder diaries, and rating scales can be used to determine the symptom diagnosis and severity [4, 5]. Symptoms of SUI are involuntary leakage during physical exertion or effort, or while sneezing or coughing. UUI is defined as involuntary leakage with an abrupt feeling of a sudden compelling desire to void that is difficult to defer. MUI is a combination of the two [6]. Pelvic floor muscle training (PFMT), in combination with lifestyle changes, is the recommended and effective first-line treatment of UI. PFMT increases the likelihood of curing UI by about eight times in women with SUI (56.1% vs 6.0%, RR = 8.38, 95% CI = 3.7–19.1) [7]. Trials of women with any type of UI report that PFMT is also more likely to cure or improve UI compared with controls [7]. Adherence to PFMT is critical for effectiveness, but compliance often decreases over time [8].

A systematic review of the perception of UI patients showed that effective treatment options are not well known, symptoms are often self-minimized and patients experience unreliable or unprofessional behavior in health professionals [9]. These factors might explain the low help-seeking numbers (about 1 in 4) in women with UI [10].

Avoidance, limiting behavior, and social embarrassment impact the quality of life of women with UI. The reduction in quality of life has been associated with severity of leakage, which is also the most important predictor of bother [11].

Depression and anxiety are two common widespread mental health disorders. A Swedish population-based survey showed a prevalence of depression of about 7% in women aged between 20 and 64 years [12]. Anxiety in the past 12 months was seen in 18% of the participants in a large American study based on face-to-face interviews. The diagnosis of anxiety was based on Diagnostic and Statistical Manual of Mental Disorders, 4th Edition criteria for a variety of anxiety disorders [13]. A large Norwegian population-based study showed that middle-aged women with UI were more likely to have depression and anxiety than women without UI. The strongest association was seen for MUI and UUI and for severe incontinence [14].

eHealth is a new strategy for improving healthcare locally, regionally, and worldwide by using information and communication technology, e.g., through a computer or smartphone apps, for the management or prevention of diseases [15]. It offers new, flexible, and accessible treatment options and it has been proven that attendance rates can increase through the use of apps or a text message reminder [16]. Young age, high level of education, female sex, and high income are all predictors that increase the likelihood of engaging in eHealth activities [17]. eHealth programs, both Internet- and app-based, aimed at women with SUI, have shown effectiveness in terms of the symptoms of incontinence, quality of life, and number of leakages, both in the short and the long term [18–20]. Increased access to first-line treatment and adherence to PFMT are possible outcomes of expanded eHealth-mediated treatment [19, 20]. An RCT study on the first-line app treatment of UUI/MUI was completed in 2018, whereby the effect of self-management with the support of an app, including programs for PFMT, bladder training, and psychological education, was compared with an information app.

The aims of this study were to assess the prevalence of anxiety and depression in women with UI who had turned to eHealth for treatment, to identify factors associated with anxiety and depression in this population, and to compare our findings with data from studies based on a general population.

## Materials and methods

The material in this study is based on the data from two RCT studies within the eContinence project registered at clinicaltrials.gov (ID: NCT01032265, NCT03097549).

**Fig. 1:**
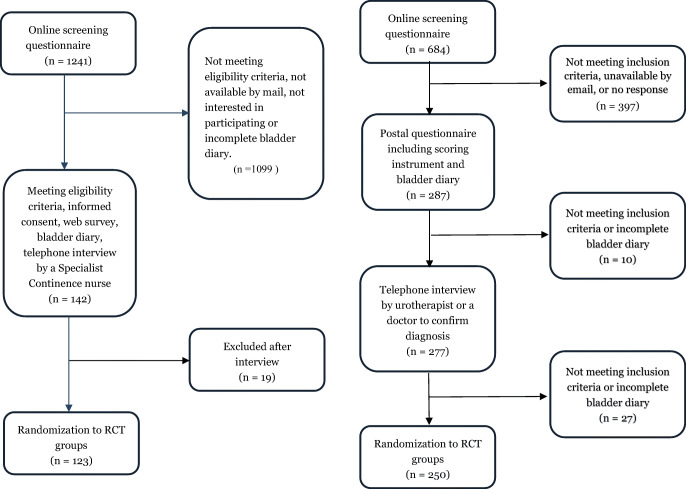
Flow chart of the stress urinary incontinence and mixed urinary incontinence/urgency urinary incontinence study.* RCT* randomized controlled trial

Between 2009 and 2011, an RCT study to evaluate the effectiveness of Internet-based PFMT in 250 women with SUI was conducted by the eContinence group at Umeå University, Sweden. The enrolment process is shown in Fig. 1. Advertisements for the recruitment of participants were published in two well-known Swedish newspapers, “Dagens Nyheter” and “Metro.” First, an initial screening survey was answered at http://www.econtinence.se, which was used to assess eligibility criteria (Table 1). No participant was excluded because of a Hospital Anxiety and Depression Scale (HADS) of >15. Informed consent, a bladder diary, and a more extensive questionnaire were completed by those who were eligible and interested in participating in the study. The ICIQ-UI-SF is a validated questionnaire that grades how often, how much, and how leakage affects everyday life [21]. Finally, participants were interviewed by a specialist continence nurse (urotherapist) by phone, to confirm the SUI diagnosis. If symptoms or problems existed that needed further assessment, the participant was encouraged to seek ordinary care and was excluded from the study [20].

**Table 1:** Inclusion and exclusion criteria in the two studies of women with stress urinary incontinence (SUI) and urgency or mixed urinary incontinence (UUI and MUI)

Inclusion criteria SUI and UUI/MUI	Exclusion criteria SUI and UUI/MUI
Common criteria	
Female	Pregnancy
Ability to read and write Swedish	Previous incontinence surgery
Computer/smartphone access	Known malignancy in lower abdomen
	Macroscopic hematuria
	Intramenstrual bleedings
	Decreased sensibility in the lower abdomen or legs
	Difficulties in emptying the bladder
Only in UUI/MUI	
Age 18 years or older	Duration of urgency symptoms <12 months
At least two leakages per week	History of febrile urinary tract infection
	History of recurrent cystitis
	Painful micturition or urgency
	Diseases such as MS, Parkinson’s, previous stroke or diabetes
Only in SUI	
At least one leakage per week	Severe psychiatric disorders, or HADS-A or HADS-D > 15
Age 18–70 years	

*HADS* Hospital Anxiety and Depression Scale

The recruitment for the second RCT study took part during 2017 and 2018. This study aimed to evaluate the efficacy of self-management of MUI or UUI in women, with the support of a new app, Tät II. Recruitment to this study took place via press releases resulting in media coverage on radio and television, and also through two Facebook campaigns aimed at women aged between 30 and 80 years. The enrolment was similar to that of the first study (see Fig. 1) in terms of initial screenings, bladder diaries, scoring instruments, and interviews by a doctor or urotherapist. Inclusion and exclusion criteria were also similar for both studies (see Table 1). Data from questionnaires about age, educational level, BMI, presence of chronic disease, HADS, ICIQ-UI-SF, and type of incontinence were used.

Screening for anxiety and depression at baseline was carried out using the HADS [22]. The scale was developed to identify possible cases of anxiety and depression through a structured and easy-to-use questionnaire. The HADS consists of two subscales of 14 assorted questions: 7 questions measuring anxiety (HADS-A) and 7 measuring depression (HADS-D). Each question has four possible answers and a score between 0 to 3 is given. The total score is then calculated and can range between 0 and 21 points on each subscale with a higher score indicating more severe symptoms. The threshold score for “possible cases” of both anxiety and depression is 8 points or more, which is the threshold identified to give the optimal balance between specificity and sensitivity. This was noticed in a large literature review where the sensitivity and specificity were approximately 0.80 for both HADS-A and HADS-D [23]. A cut-off score of ≥8 was used to compare the prevalence of anxiety and depression in women with different kinds of UI in this study.

The presence of a disease or treatment for a disease was measured using a question asking the participants if they had been (in the SUI study) or were being (in the UUI/MUI study) treated for high blood pressure, heart disease, asthma, anxiety or depression, kidney disease, cancer, or other long-term disease. If a woman had at least one of the medical conditions she was classified as having a disease or being treated for a disease.

With regard to statistical analysis, as a first step, descriptive statistics with baseline characteristics for SUI and UUI/MUI participants were presented, and Chi-squared tests and* t* tests were used to analyze differences between groups. Baseline characteristics included age, educational level categorized as university education (yes/no), BMI (≤25/>25), anxiety or depression (as described above), ICIQ-UI-SF score, severity of UI (“slight” 1–5 points, “moderate” 6–12 points, “severe” 13–18 points, and “very severe” 19–21 points) [24], and the presence of disease or treatment for disease (yes/no).

Second, we analyzed the characteristics (the same as in the first step except for the severity of UI) for women with or without depression or anxiety, and used Chi-squared tests and* t* tests for differences between groups.

Third, we analyzed the odds of having depression using the logistic regression technique with 95% confidence intervals. Both unadjusted and adjusted analyses were performed, and the final model included age, BMI, ICIQ-UI-SF score, type of incontinence, and disease or treatment. As the odds ratio for type of UI increased between the unadjusted and adjusted model, an interaction between UI type and disease/treatment was suspected. An interaction term was therefore included, but as no interaction was seen, the term was excluded from the final model.

All statistical analyses were performed using the statistical software SPSS version 25.

This study was approved by the Regional Ethical Review Board, Umeå (08-124 M and 2016/523–31) and by the Regional Ethical Board, Uppsala (2019–03789).

## Results

Of the 684 women screened for the SUI study, 250 were included in this study and of the 1,241 women screened for the MUI/UUI study, 123 were finally included (Fig. 1). Only baseline data were analyzed.

Women with UUI or MUI were older than women with SUI, mean age 58.3 vs 48.6 years (*p* = <0.001). To a larger extent, they also had a university education, 85.4% vs 75.2% (*p* = 0.025). More women in the MUI/UUI group were overweight (*p* = 0.001). They also had a higher mean HADS-D score (*p* = 0.004) and more severe UI according to the ICIQ-UI-SF (*p* = 0.001) than women with SUI. About 4 in 10 women with UUI or MUI had symptoms classified as severe or very severe compared with about 1 in 4 women with SUI (Table 2).

**Table 2:** Characteristics of 250 women with stress urinary incontinence (SUI) and 123 women with urgency or mixed urinary incontinence (UUI/MUI)

	SUI (*n* = 250)	UUI/MUI (*n* = 123)	*p* value
Age, mean (SD)	48.6 (10.2)	58.3 (9.6)	<0.001*
University education,* n* (%)	188 (75.2)	105 (85.4)	0.025**
BMI > 25,* n* (%)	84 (33.6)	64 (52.0)	0.001**
HADS-A ≥8,* n* (%)	31 (12.4)	17 (13.8)	0.700**
HADS-D ≥8,* n* (%)	8 (3.2)	13 (10.6)	0.004**
HADS-A, mean (SD)	3.6 (3.0)	3.9 (3.5)	0.452*
HADS-D, mean (SD)	2.2 (2.3)	3.2 (3.2)	0.001*
ICIQ-UI-SF, mean (SD)	10.4 (3.3)	11.6 (3.3)	0.001*
Severity of UI			
Slight 1–5,* n* (%)	14 (5.6)	3 (2.4)	
Moderate 6–12,* n* (%)	170 (68.0)	73 (59.3)	
Severe 13–18,* n* (%)	64 (25.6)	43 (35.0)	
Very severe 19–21,* n* (%)	2 (0.8)	4 (3.3)	
Disease or treatment,* n* (%)	93 (37.5)^a^	46 (37.4)	0.985**

*HADS* Hospital Anxiety and Depression Scale, *ICIQ-UI-SF* International Consultation on Incontinence Questionnaire Urinary Incontinence-Short Form

*p value by* t* test

**p value by Chi-squared test

^a^Two missing in SUI

There was no difference in the prevalence of anxiety between groups. Depression was significantly more common in women with UUI or MUI compared with women with SUI (10.6% vs 3.2%;* p* = 0.004; see Table 2). For all women with UI regardless of type, the prevalence of depression was 5.6% and that of anxiety was 12.7%.

In Table 3, the characteristics are presented in relation to the presence of depression or anxiety. The mean age, level of education, and the severity of incontinence did not differ between women with or without anxiety or depression. Women with depression stated significantly more often that they had a chronic disease or were treated for a chronic disease. There was a higher tendency for women with depression to be overweight (BMI >25), but these data did not reach statistical significance.

**Table 3:** Characteristics and comparisons between participants with and without symptoms of depression and anxiety

	Depression (*n* = 21)	Without depression (*n* = 352)	*p* value	Anxiety (*n* = 48)	Without anxiety (*n* = 325)	*p* value
Age, mean (SD)	53.7 (10.7)	51.7 (11.0)	0.426*	50.4 (11.6)	52.0 (10.9)	0.336*
ICIQ-UI-SF score^a^, mean (SD)	11.9 (3.3)	10.7 (3.4)	0.116*	11.4 (3.3)	10.7 (3.4)	0.163*
BMI >25,* n* (%)^b^	12 (60.0)	136 (38.6)	0.058**	20 (42.6)	128 (39.4)	0.678**
University education,* n* (%)	17 (81.0)	276 (78.4)	0.783**	39 (81.3)	254 (78.2)	0.626**
Disease or treatment,* n* (%)^c^	14 (66.7)	125 (35.7)	0.004**	26 (54.2)	113 (35.0)	0.004**

*p value by* t* test

**p value by Chi-squared test

^a^International Consultation on Incontinence Questionnaire Urinary Incontinence-Short Form (ICIQ-UI-SF) measuring symptoms

^b^One person is missing within BMI >25

^c^Two missing in stress urinary incontinence

In the final adjusted analyses, we found that the odds of depression increased four-fold for women with UUI/MUI (OR 4.2 [95% CI 1.4–12.3]) compared with those with SUI. Women with a chronic disease had increased odds of 3.6 (95% CI 1.4–9.2) for depression compared with women without chronic disease. The odds were adjusted for age, severity of incontinence, and BMI (Table 4).

**Table 4:** Risk factors of symptoms of depression^a^ in 373 women with urinary incontinence

	Unadjusted OR	95% CI	*p* value	Adjusted OR	95% CI	*p* value*
Age	1.02	0.98–1.06	0.427	0.98	0.94–1.03	0.510
ICIQ-UI-SF score ^b^	1.11	0.98–1.26	0.118	1.05	0.90–1.20	0.582
BMI > 25 vs ≤25^c^	2.38	0.95–5.98	0.064	1.52	0.56–4.12	0.408
UUI/MUI vs SUI^d^	3.56	1.44–8.87	0.006	4.19	1.43–12.30	0.009
Disease or treatment	3.60	1.42–9.15	0.007	3.33	1.24–8.92	0.017

*p value by binary logistic regression, adjusted OR. The odds were adjusted for age, severity of incontinence, and BMI

^a^Depression defined as 8 points or more on the Hospital Anxiety and Depression scale (HADS)

^b^Symptom severity evaluated using the International Consultation on Incontinence Modular Questionnaire Urinary Incontinence-Short form (ICIQ-UI-SF)

^c^One person is missing within BMI > 25

^d^Urgency or mixed urinary incontinence (UUI/MUI) versus stress urinary incontinence (SUI)

## Discussion

In this eHealth study, in which one third of the women had severe or very severe incontinence, we found considerably lower prevalence of anxiety and depression than previously reported in large population-based studies of women with UI. In the adjusted analyses, the odds of having depression were four-fold for women with UUI or MUI compared with SUI, and three-fold for women with a chronic disease when healthy or untreated women were the reference.

The strengths of this study include the large numbers of participants with incontinence confirmed by interview during the extensive enrolment, the participants’ broad age range (from 23 to 77 years), and the ability to include many known risk factors in the analyses. There were very few missing values in the multivariate analyses. The validated measurements the HADS and the ICIQ-UI SF were used [21, 23], making it possible to compare ours with other large studies of anxiety and depression.

One of the limitations of this study is that there were comparatively few participants with depression or anxiety, making analyses of subgroups difficult. Another limitation is the difference in the formulation of the questions about disease or treatment in the initial questionnaire; “I don’t have any chronic disease” in the SUI questionnaire, contra “I don’t have any treatment for any chronic disease.” For the analysis of factors associated with depression, it is possible that factors other than those for which we had data might be of importance. The second study started 6 years after the first study and we cannot rule out that the increased use of eHealth during this period might have influenced our results.

Two large population-based surveys using the HADS and the same definition of UI had previously found a higher prevalence of anxiety (22.9–30.4% [SUI], 28.1–30.2% [UUI], and 32.0–49.1% [MUI]) and depression (9.0–16.8% [SUI], 11.7–17.8% [UUI], and 16.9–34.7% [MUI]). These two studies also used a HADS-A or HADS-D score of ≥8 as the threshold for defining anxiety or depression [14, 25]. In our study the corresponding prevalence of anxiety was 12.4% (SUI) and 13.8% (MUI/UUI) and the prevalence of depression was 3.2% (SUI) and 10.6% (MUI/UUI).

Compared with 5.4% of severe or very severe incontinence in the Norwegian study referred to [14], 26.4% of women with SUI and 38.3% of women with UUI/MUI had severe or very severe incontinence in our study. We found a lower prevalence of both anxiety and depression, even though a higher severity has previously been associated with higher levels of anxiety and depression [14]. There were some differences between studies in terms of mean age, but the studies controlled for this factor [14] or used weighted sampling methods [25].

One reason for the comparatively low prevalence we found in our study could be the high level of education of our population, reflecting a high socioeconomic status. There is an association between anxiety and depression and a low level of education [26]. eHealth usage is also extensive among highly educated women as a group, and the lowest usage of the internet is seen in old people and in groups with a low level of education [17, 26]. The disparity in incidence and burden of diseases is large between the groups. Low income, gender, level of education, and ethnicity are all social factors that influence health. Differences in the usage of eHealth between socioeconomic groups have been identified as an important factor in the implementation and development of eHealth [27] and we need to know more about how to develop and implement eHealth without increasing social health inequalities [17, 28]. Access to apps and the Internet is elementary in the usage of eHealth and these technologies are widespread nowadays: in 2018, 90% of the Swedish population owned a smartphone and 95% used the Internet [29]. Even though Internet access is high in Sweden, Internet use itself and the type of use vary widely based on age and educational status [29].

Other reasons for the prevalence rates we found could be: the lower propensity to use eHealth among people with anxiety and depression, embarrassment or evasion, and also a lower ability to attend research studies [30]. Women in our study took an active decision to seek treatment for UI; this may also affect symptoms of anxiety or depression.

We found a four-fold increase in the odds of having depression in women with MUI/UUI compared with SUI. In population-based studies of women with UI, depression prevalence is highest in MUI and lowest in SUI and this is in line with our findings [14, 25]. In the Norwegian study, UI was found to be associated with both depression and anxiety, with the strongest associations for MUI/UUI [14].

In cross-sectional population-based studies and in studies like ours, it is not possible to investigate causality. We do not know whether depression could be a consequence of UUI /MUI, whether UI could be a consequence of depression, or whether the symptoms just coexist because of biological, psychological, or environmental factors. Leakage with a sense of urgency has a larger impact on quality of life, as the leakage is more unpredictable and may therefore cause isolation and depression [11].

## Conclusion

We found increased odds of having depression in women with MUI/UUI compared with those with SUI. Yet, the prevalence of anxiety and depression among women who turned to eHealth for treatment was considerably lower than that reported in large cross-sectional surveys. This may partly be explained by differences between studies in terms of socioeconomic factors. The use of eHealth is still more common in highly educated populations and we need to investigate barriers and identify measures that may increase access to and use of eHealth.
